# Supplementary material for: Resolving the full spectrum of human genome variation using Linked-Reads
Source: Genome Res. 2019 Apr;29(4):635–45. doi: 10.1101/gr.234443.118 (PMC6442396; doi:10.1101/gr.234443.118)
Supplement: Supplemental Material [file supp_gr.234443.118_Supplemental_longranger-2.2.zip › longranger-2.2.tar.bz2!/lr2.2-public/lib/stan/cmdstan/stan/doc/api/html/structstan_1_1model_1_1model__functional.html]

Stan: stan::model::model\_functional< M > Struct Template Reference


|  |  |
| --- | --- |
|  | Stan  2.5.0  probability, sampling & optimization |


- Main Page
- Related Pages
- Namespaces
- Classes
- Files

- Class List
- Class Index
- Class Hierarchy
- Class Members

All Classes Namespaces Files Functions Variables Typedefs Enumerations Enumerator Friends Macros Pages

- stan
- model
- model\_functional

Public Member Functions |
Public Attributes |
List of all members

stan::model::model\_functional< M > Struct Template Reference

`#include <util.hpp>`

|  |  |
| --- | --- |
| Public Member Functions | |
|  | model\_functional (const M &m, std::ostream \*out) |
|  | |
| template<typename T > | |
| T | operator() (Eigen::Matrix< T, Eigen::Dynamic, 1 > &x) const |
|  | |

|  |  |
| --- | --- |
| Public Attributes | |
| const M & | model |
|  | |
| std::ostream \* | o |
|  | |

## Detailed Description

### template<class M> struct stan::model::model\_functional< M >

Definition at line 390 of file util.hpp.

## Constructor & Destructor Documentation

template<class M >

|  |  |  |  |
| --- | --- | --- | --- |
| stan::model::model\_functional< M >::model\_functional | ( | const M & | *m*, |
|  |  | std::ostream \* | *out* |
|  | ) |  |  |

inline

Definition at line 395 of file util.hpp.

## Member Function Documentation

template<class M >

template<typename T >

|  |  |  |  |  |  |
| --- | --- | --- | --- | --- | --- |
| T stan::model::model\_functional< M >::operator() | ( | Eigen::Matrix< T, Eigen::Dynamic, 1 > & | *x* | ) | const |

inline

Definition at line 398 of file util.hpp.

## Member Data Documentation

template<class M >

const M& stan::model::model\_functional< M >::model

Definition at line 392 of file util.hpp.

template<class M >

std::ostream\* stan::model::model\_functional< M >::o

Definition at line 393 of file util.hpp.

---

The documentation for this struct was generated from the following file:

- src/stan/model/util.hpp


---

[ Stan Home Page ]

*© 2011–2014,
Stan Development Team.*
